# The ABC transporter Pdr18 is required for yeast thermotolerance due to its role in ergosterol transport and plasma membrane properties

**DOI:** 10.1111/1462-2920.15253

**Published:** 2020-10-11

**Authors:** Cláudia P. Godinho, Rute Costa, Isabel Sá‐Correia

**Affiliations:** ^1^ iBB ‐ Institute for Bioengineering and Biosciences, Instituto Superior Técnico, Universidade de Lisboa Lisbon Portugal; ^2^ Department of Bioengineering Instituto Superior Técnico, Universidade de Lisboa Lisbon Portugal

## Abstract

Among the mechanisms by which yeast overcomes multiple stresses is the expression of genes encoding ATP‐binding cassette (ABC) transporters required for resistance to a wide range of toxic compounds. These substrates may include weak acids, alcohols, agricultural pesticides, polyamines, metal cations, as in the case of Pdr18. This pleotropic drug resistance transporter was previously proposed to transport ergosterol at the plasma membrane (PM) level contributing to the maintenance of PM lipid organization and reduced diffusional permeation induced by lipophilic compounds. The present work reports a novel phenotype associated with the putative drug/xenobiotic‐efflux‐pump transporter Pdr18: the resistance to heat shock and to long‐term growth at supra‐optimal temperatures. Cultivation at 40°C was demonstrated to lead to higher PM permeabilization of a *pdr18*Δ cell population with the *PDR18* gene deleted compared with the parental strain population, as indicated by flow cytometry analysis of propidium iodide stained cells. Cells of *pdr18*Δ grown at 40°C also exhibited increased transcription levels from genes of the ergosterol biosynthetic pathway, compared with parental cells. However, this adaptive response at 40°C was not enough to maintain PM physiological ergosterol levels in the population lacking the Pdr18 transporter and free ergosterol precursors accumulate in the deletion mutant cells.

## Introduction

Microbial adaptation to environmental stresses is considered as one of the most challenging topics of biological research and, in biotechnology, the mechanistic understanding of adaptation to environmental stress is essential to construct superior industrial strains (Sá‐Correia, 2019). The eukaryotic model *Saccharomyces cerevisiae* is the most widely used yeast species in fermentation industries but shows decreased fermentation efficiency at temperatures above 35°C due to the conjugated inhibitory effect of ethanol, acetic acid and other chemical stresses present in the industrial setting at high temperatures (van Uden and da Cruz Duarte, 1981; Sá‐Correia and Van Uden, 1983; Choudhary *et al*., 2016). In fact, the presence of those chemical stresses leads to the decrease of the maximal and optimal temperatures for growth (Sá‐Correia and Van Uden, 1983; Ramos and Madeira‐Lopes, 1990). Growth at supra‐optimal temperatures implicates increased fluidity of cell membranes, affects protein folding and stability and disturbs cytoskeleton structures, ultimately leading to metabolic imbalances and, depending on the level of thermal stress, to decreased specific growth rate or loss of viability (Riezman, 2004; Verghese *et al*., 2012; Woo *et al*., 2014). The impact of supraoptimal temperatures in cellular membranes is of major importance, since the induced fluidification may lead to its decreased/loss of function as a selective barrier and home for embedded proteins involved in major physiological roles (Henderson and Block, 2014; Schuberth and Wedlich‐Söldner, 2014; Godinho *et al*., 2018a). Plasma membrane permeabilization is responsible for increased passive proton influx and consequent intracellular acidification when cells are in acidic media, higher diffusion rate of membrane‐permeant compounds and even of otherwise membrane‐impermeant toxicants (van Uden and da Cruz Duarte, 1981; Weitzel *et al*., 1987; Piper, 1993; Godinho *et al*., 2018a; Lindahl *et al*., 2018). The adaptation of yeast cells to higher temperatures involves the reprogramming of gene expression favouring the rearrangement of plasma membrane by altering lipid composition and organization, the synthesis of heat shock proteins (HSPs), among other responses (Suutari *et al*., 1990; Riezman, 2004; Richter *et al*., 2010; Verghese *et al*., 2012). Several HSPs are involved in plasma membrane structure protection, such as it is the case of the integral plasma membrane Hsp30, hypothesized to regulate or stabilize plasma membrane components (Panaretou and Piper, 1992; Régnacq and Boucherie, 1993). These adaptive responses require significant energy consumption and are non‐inheritable, resulting from the activation of specific stress‐related genes. Most of the yeast strains reported as intrinsically thermotolerant have been isolated from bioethanol production plants where they were exposed to high temperatures for long periods of time, but the genome‐wide characterization of these strains is poorly described (Amorim *et al*., 2010; Abreu‐Cavalheiro and Monteiro, 2013; Hamouda *et al*., 2016; Kechkar *et al*., 2019). Based on adaptive laboratory evolution experiments, mutations that abrogated the expression of the C‐5 sterol desaturase Erg3, a non‐essential ergosterol biosynthetic enzyme, were found to lead to thermotolerance by replacement of the ‘flat‐structured’ ergosterol with the ‘bend‐structured’ fecosterol in the plasma membrane (Caspeta *et al*., 2014). Moreover, the lack of *ERG5* expression, encoding a C‐22 sterol desaturase, rendered yeast cells more tolerant to growth at 39.5°C (Liu *et al*., 2017). These studies point to the engineering of plasma membrane sterol composition as an important target for the improvement of *S*. *cerevisiae* thermotolerance.

Among other mechanisms, the acquisition of resistance to multiple stresses is mediated by plasma membrane proteins of the ABC (ATP‐binding cassette) or the major facilitator superfamily (MFS) superfamilies proposed to catalyse the extrusion of the corresponding toxic compounds (Godinho and Sá‐Correia, 2019). The present study is focused on the Pdr18 transporter belonging to the ABC superfamily and previously confirmed to be involved in multidrug/multixenobiotic resistance (MDR/MXR) (Godinho and Sá‐Correia, 2019). In line with accumulating evidence supporting the notion that yeast ABC proteins perform biological activities other than their accepted role as drug exporters, among them the transport of lipids (dos Santos *et al*., 2014; dos Santos and Sá‐Correia, 2015; Prasad *et al*., 2016; Godinho and Sá‐Correia, 2019), Pdr18 was proposed to be responsible for the active transport of ergosterol at the plasma membrane level (Cabrito *et al*., 2011; Godinho *et al*., 2018a). Ergosterol concentration in the plasma membrane is essential to counteract acetic acid and the herbicide 2,4‐dichlorophenoxyacetic acid (2,4‐D) stress‐induced permeabilization and reduce the passive diffusion rate of the undissociated toxic forms of these weak acids (Cabrito *et al*., 2011; Godinho *et al*., 2018a). The expression of *PDR18* also confers yeast with tolerance to a very wide range of chemical compounds, several of them of industrial relevance (Teixeira *et al*., 2012; Godinho *et al*., 2018b). In the present study, we report, for the first time, the involvement of Pdr18 in short‐ and long‐term adaptation of yeast growth at supra‐optimal temperatures. The underlying mechanisms are proposed as well as *PDR18* as an interesting candidate for genome engineering towards improved yeast thermotolerance among other stresses of relevance in industrial bioprocesses.

## Results

### Pdr18 is a thermotolerance determinant leading to decreased heat‐induced plasma membrane permeabilization

**Fig 1:**
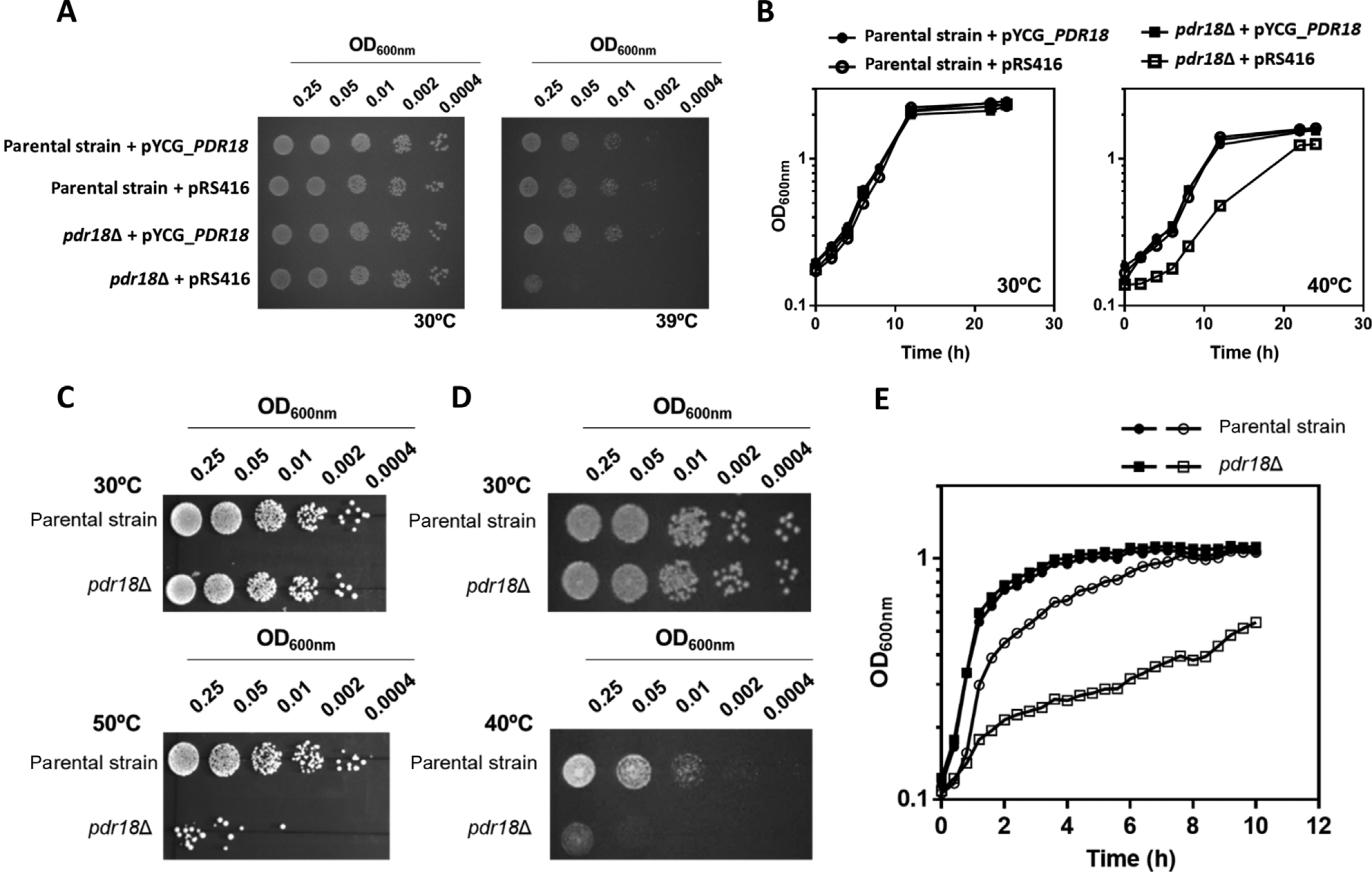
Susceptibility of the parental and *pdr18*Δ strains to supra‐optimal temperatures. A. Complementation assays of growth in solid MM4‐U medium of the parental and *pdr18*Δ strains, harbouring plasmid pRS416 (cloning vector) or the recombinant vector expressing *PDR18* from its natural promoter (pYCG_*PDR18*), incubated at 30°C or at 39°C.B. Complementation assays of growth in liquid MM4‐U media pH 4.5, for the parental (●,○) and *pdr18*Δ strains (■,□), harbouring pRS416 (○,□) or the derived pYCG*_PDR18* (●,■) incubated at 30°C or at 40°C. C. Comparison of growth by spot assays of the parental strain and *pdr18*Δ mutant cell suspensions plated in solid YPD media following 30 min incubation either at 30 or at 50°C. D. Comparison of growth by spot assays of the parental strain and *pdr18*Δ mutant cell suspensions plated in solid YPD media and incubated for 48 h either at 30 or at 40°C. E. Growth curves of the parental (●,○) and *pdr18*Δ (■,□) strains in YPD liquid medium at pH 4.5, incubated at 30°C (●,■) or 40°C (○,□), based on culture OD_600nm_. Results of all panels of the figure are representative of at least three independent growth experiments.

The expression of the *PDR18* gene was found to be a determinant of yeast tolerance to short‐term heat shock and long‐term cultivation at supra‐optimal temperatures (Fig. 1). The role of Pdr18 as a determinant of yeast thermotolerance was observed by spot assays in agar plates of MM4‐U medium incubated at 39°C (Fig. 1A) and by cultivation in liquid MM4‐U medium incubated at 40°C (Fig. 1B). Complementation assays of the susceptibility phenotype of *pdr18*Δ at 39°C and 40°C confirmed the role of *PDR18* as a thermotolerance determinant since the transformation of the mutant with a centromeric plasmid expressing the *PDR18* gene under the control of its natural promoter rescued the parental strain phenotype (Fig. 1A and B). However, the expression of Pdr18 from the same recombinant plasmid in the parental strain did not further improve its thermotolerance (Fig. 1A and B). Therefore, the continuation of this work in Yeast‐extract Peptone Dextrose (YPD) media was focused on the parental strain and the derived deletion mutant *pdr18*Δ.

A 30‐min' exposure to 50°C was found to lead to a marked loss of viability of the *pdr18*Δ deletion mutant population, when compared with the parental strain population (Fig. 1C). Moreover, the parental strain population adapts and grows at 40°C more efficiently when compared with the *pdr18*Δ population (Fig. 1D). The role of Pdr18 in growth at supra‐optimal temperatures was confirmed by yeast cultivation in liquid YPD medium (using a starting OD_600nm_ of 0.1). While no significant differences were observed for the growth curves of both strains at 30°C, growth at 40°C exhibited a reduced growth rate with a more dramatic reduction for the mutant with the *PDR18* deleted (Fig. 1E). As described by van Uden's laboratory for yeasts with associative temperature profiles, like *S*. *cerevisiae*, the growth curves at the supraoptimal temperature of 40°C exhibit two periods of exponential growth (Van Uden, 1985). The first period corresponds to the first phase of growth with a higher maximum specific growth rate during which thermal death is not registered (first 2 h of cultivation; Fig. 1E); it is followed by a second period of balanced exponential growth with reduced growth kinetics presumably as a consequence of the simultaneous occurrence of exponential growth and death (Van Uden, 1985).

**Fig 2:**
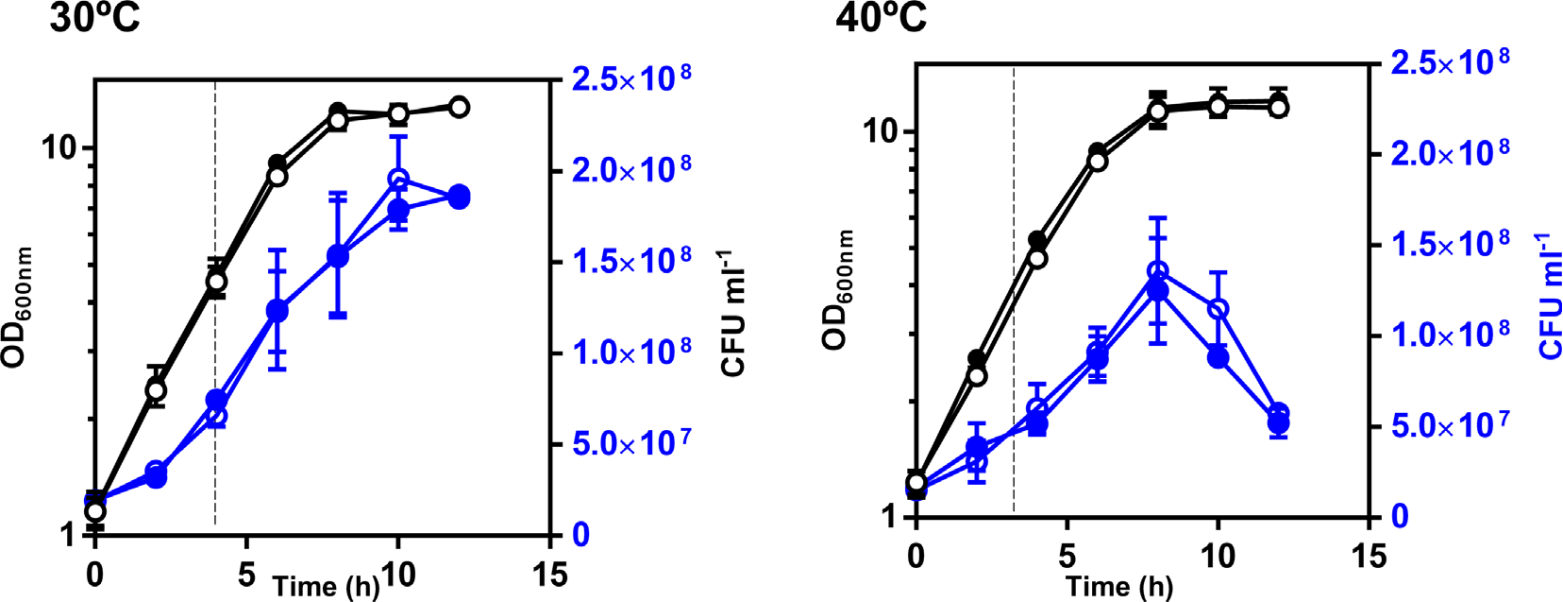
Growth and viability of the parental and *pdr18*Δ strains populations in YPD media, at 30°C or 40°C. Yeast strains' performance was compared by following OD_600nm_ (black) and CFU ml^−1^ (blue) during cultivation at 30°C and 40°C. The parental strain is represented by the open symbols and the *pdr18*Δ by the closed symbols. Dashed lines indicate the time points at which cells were harvested for further analyses. Results are means of three independent experiments and error bars represent standard deviation.

The impact on growth of the supra‐optimal growth temperature of 40°C was also assessed by cultivation in YPD medium using a higher starting OD_600nm_ of 1, compared with 0.1 used for Fig. 1 experiments, a condition closer to industrial fermentation inoculation conditions (Kapu *et al*., 2013) (Fig. 2). When such higher inoculum was used, the growth curves assessed by culture OD_600nm_ at 40°C for the mutant and the parental strains were apparently not different and the two phases of growth were difficult to identify; the second phase appears later, after around 5 h of incubation when a slight reduction of the specific growth rate was registered and the growth curve still was far from the stationary phase (Fig. 2). A detectable loss of the population viability after 8 h of cultivation was observed and this loss was more evident for the *pdr18*Δ mutant (Fig. 2).

**Fig 3:**
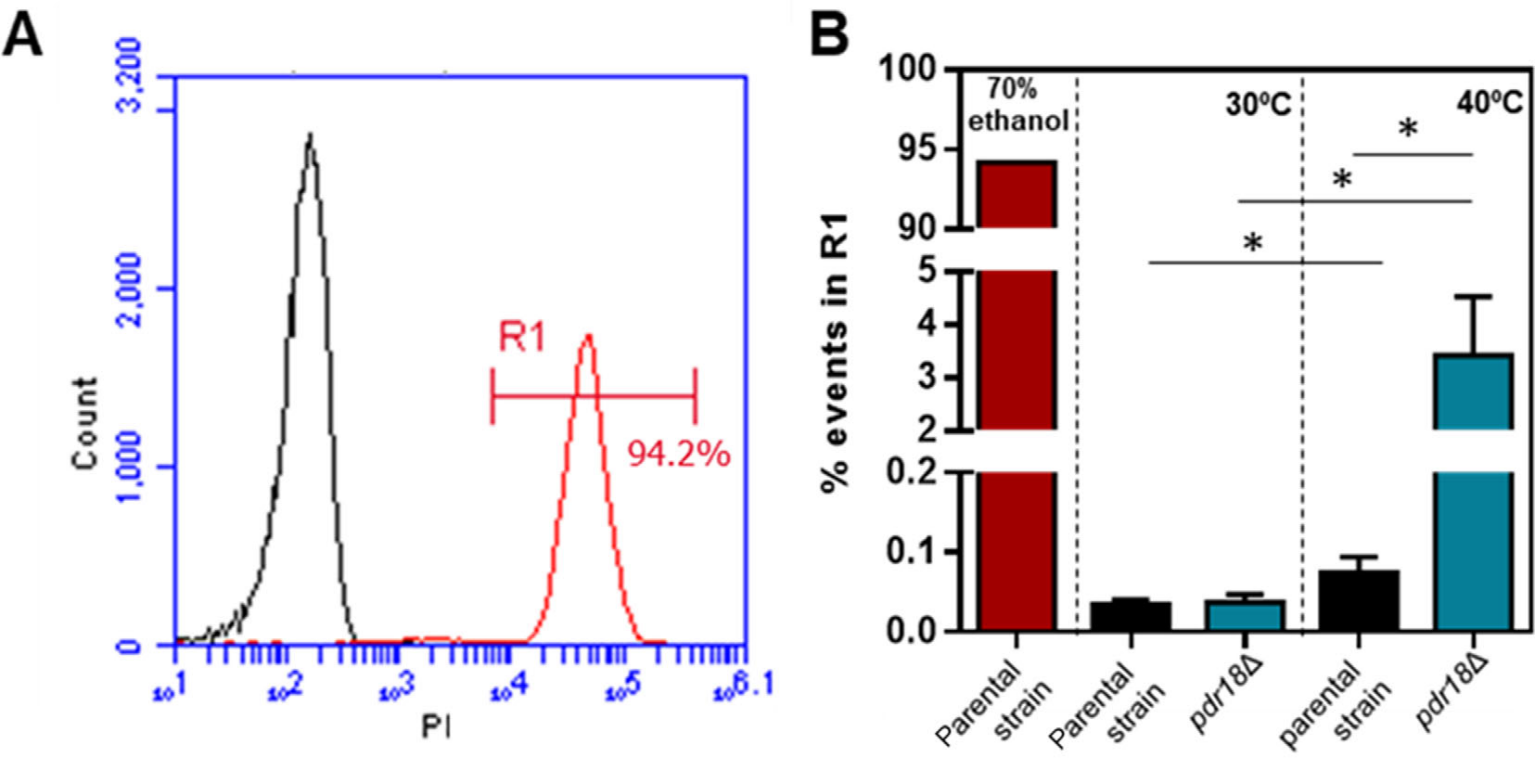
Flow cytometry analysis of plasma membrane permeability of yeast cells cultivated at 30°C and 40°C, using propidium iodide. A. PI fluorescence distribution of parental strain cells harvested from cultivation at 30°C and permeabilized (red) or not (black) with 70% (vol./vol.) ethanol. R1 represents PI‐positive region defined by the permeabilization control condition (red). B. Percentage of the population falling in the R1 region (permeabilized cells) for the conditions tested. Error bars represent standard deviation resultant from at least two biological replicates with three technical replicates each. Asterisks indicate statistical significance (*p* > 0.05; *t*‐student test).

Although the experimental conditions chosen for comparing both strains in Fig. 2 did not allow a very clear observation of the effect of 40°C versus 30°C and the differentiation of the two yeast populations, the comparison of plasma membrane permeability using propidium iodide (PI) staining and flow cytometry of cells harvested after 4.5 h of cultivation indicates a higher permeability of the cell population cultivated at 40°C versus 30°C and that the permeabilizing effect of 40°C for the yeast population deleted for *PDR18* was dramatic (Fig. 3). In these experiments, parental strain cells grown at 30°C were used as PI‐negative control, and these cells permeabilized with 70% (vol./vol.) ethanol were used as PI‐positive control (Fig. 3A in black and red respectively), allowing us to define the R1 region that encompasses the PI‐positive cells excluding living cells (Fig. 3A). Remarkably, at 40°C, 4% of the *pdr18*Δ population falls in the R1 region, compared with less than 1% in the parental strain population (Fig. 3B). PI‐positive cells are not necessarily dead and may recover from stress‐induced permeabilization (Davey and Hexley, 2011). This is corroborated by the observation that the *pdr18*Δ cell population exhibited approximately the same colony‐forming units as the parental strain, when both were cultivated at 40°C (Fig. 2).

### The transcription of *ERG* genes is upregulated in the mutant lacking *PDR18* when cultivated at 40°C

**Fig 4:**
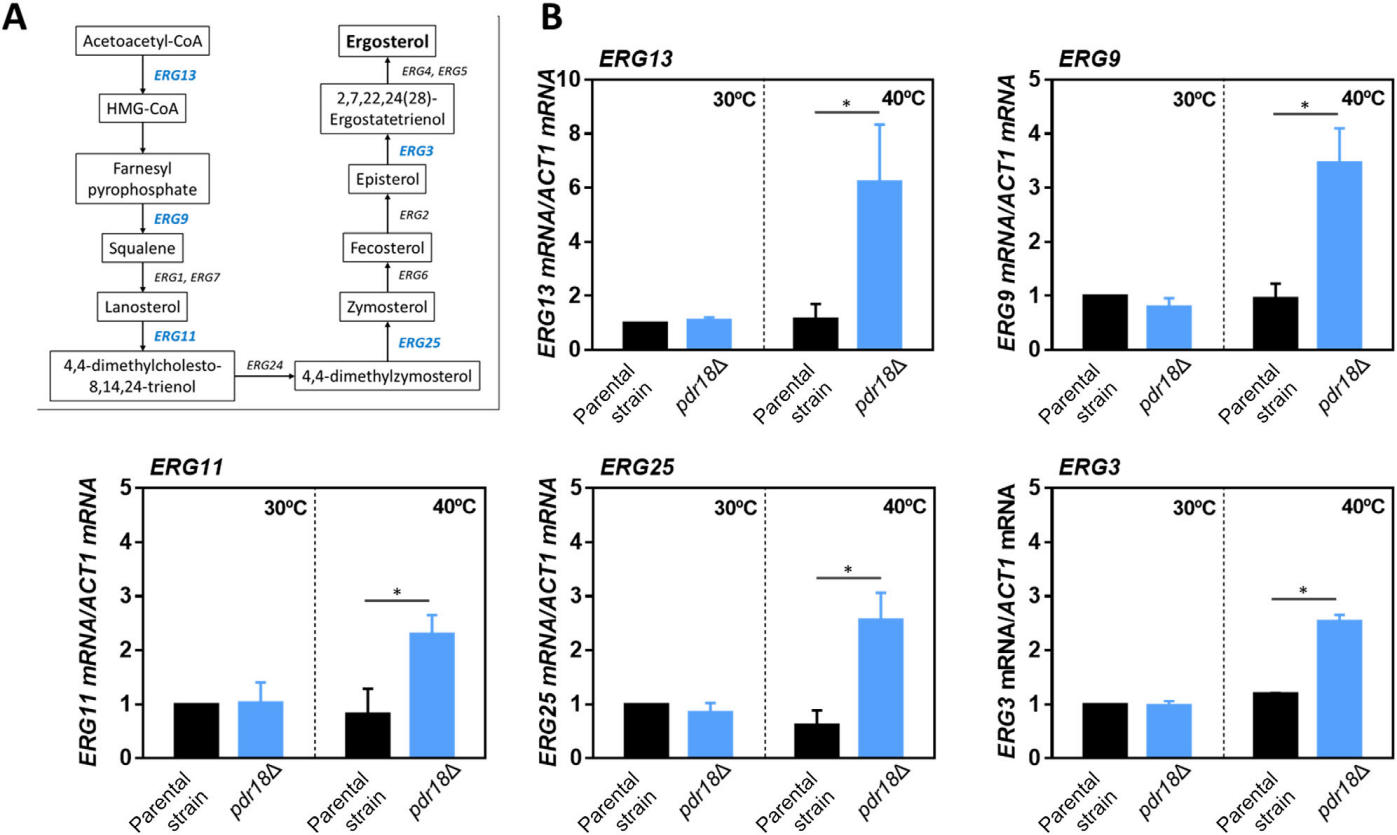
Levels of mRNA from several ergosterol biosynthetic pathway genes during cultivation in YPD, both at 30 and 40°C, of the parental and *pdr18*Δ strains. A. Ergosterol biosynthetic pathway [adapted from KEGG (http://www.genome.jp/kegg/pathway.html)] in which the *ERG* genes under study are highlighted in blue; (B) relative quantification by qRT‐PCR of the mRNA levels from the indicated genes, using *ACT1* as the reference gene. Error bars represent standard deviation resultant from at least two biological replicates with three technical replicates each. Asterisks indicate statistical significance (*p* > 0.05; *t*‐student test).

Considering the role proposed for Pdr18 (Cabrito *et al*., 2011; Godinho *et al*., 2018a), the level of transcription from genes involved in ergosterol synthesis (*ERG*) was compared in cells of the mutant *pdr18*Δ and the parental strain grown at 40°C and harvested at the same incubation time used to harvest the cells compared for permeability (Fig. 4). The transcription levels from *ERG3*, *ERG9*, *ERG11*, *ERG13* and *ERG25* genes, encoding enzymes covering the early‐, mid‐ and late‐stages of the biosynthetic pathway, were compared (Fig. 4A). The transcription from these genes has been reported to respond to environmental challenges (Gasch *et al*., 2000; Causton *et al*., 2001; Becerra *et al*., 2003; Hahn *et al*., 2004).

The transcription levels from the selected *ERG* genes were found to be similar in the parental and *pdr18*Δ strains when cells were cultivated at 30°C (Fig. 4B). However, for cells grown at 40°C, the *pdr18*Δ population exhibits significantly higher (from twofold to sixfold) transcript levels for all the *ERG* genes tested, when compared with the parental strain population levels (Fig. 4B).

### In cells devoid of Pdr18, ergosterol precursors, but not esterified sterols, are accumulated at 40°C

**Fig 5:**
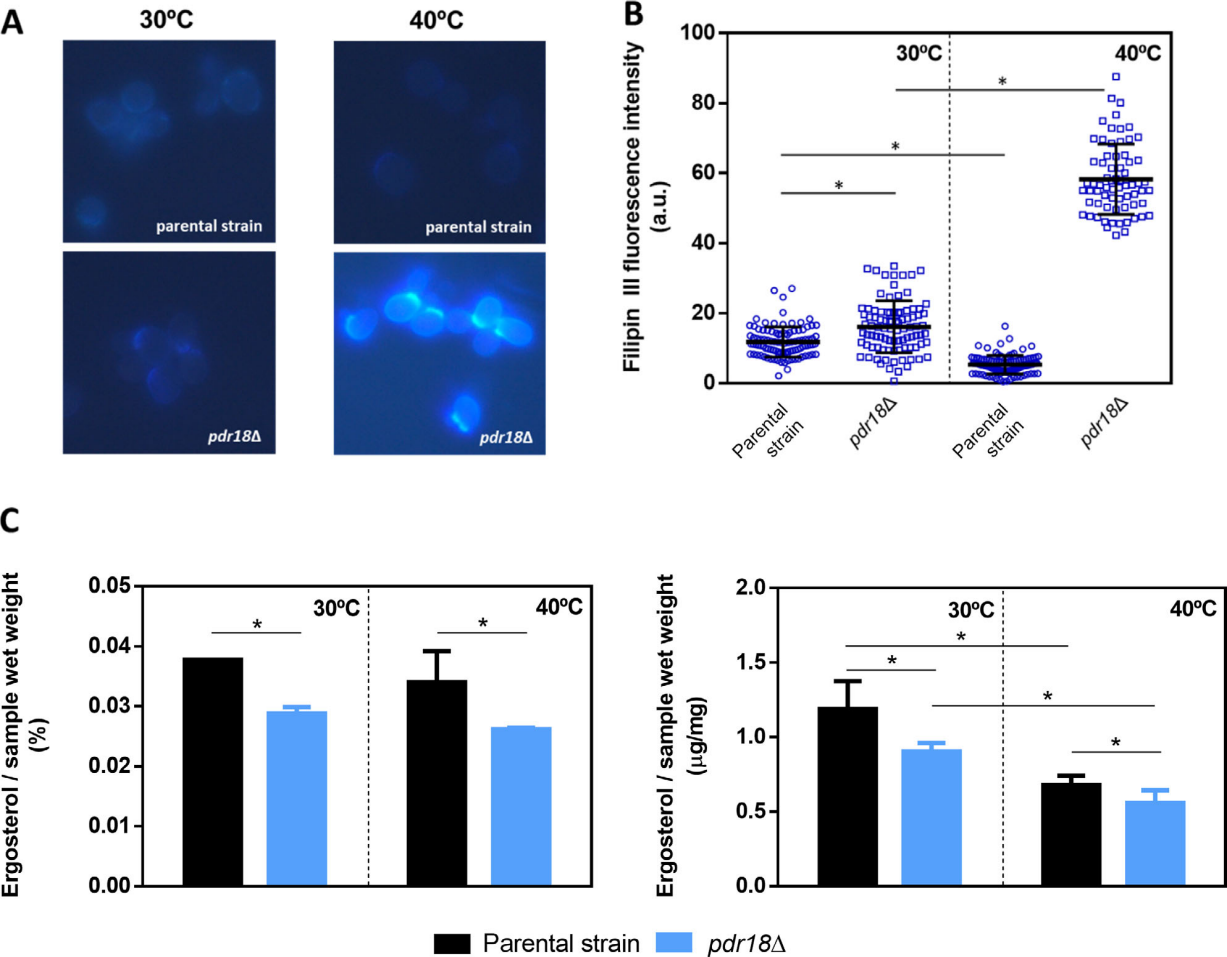
Total sterol quantification and distribution of ergosterol in the parental and *pdr18*Δ strains, during cultivation in YPD at 30°C or 40°C.A. Representative images of filipin III‐stained cells from the parental and *pdr18*Δ strains' cell populations grown at 30°C or 40°C; B. Distribution of filipin III fluorescence intensity of individual cells, in which the horizontal lines represent mean and standard deviation for each cell population; fluorescence microscopy results arise from the analysis of at least 70 cells, and all measurements were background‐corrected; C. Comparison of total cell ergosterol content of parental and *pdr18*Δ strains harvested from the indicated conditions, by two complementary methods ‐ spectrophotometric (on the left) and HPLC (on the right). The data shown represent means of three independent experiments, and error bars indicate standard deviation. Asterisks indicate statistical significance (*p* > 0.05; *t*‐student test).

The intracellular content of ergosterol and total sterols was compared in parental and *pdr18*Δ strains cells cultivated at 30°C or 40°C using the fluorescent probe filipin III. The cell populations examined were harvested in the same conditions used for permeabilization and *ERG* genes regulation experiments. Filipin III effectively binds total free sterols allowing their localization and quantification in yeast, in vivo (Beh and Rine, 2004). Illustrative images of filipin III‐stained cells are shown in Fig. 5A and the distribution of filipin III fluorescence intensity for each individual cell is shown in Fig. 5B. The results indicate that parental cells grown at 40°C exhibited lower levels of filipin III fluorescence, when compared with cells grown at 30°C, reflecting the lower total sterol content present in the parental strain cells when cultivated at that supra‐optimal temperature of growth (Fig. 5B). When cultivated at 30°C, the *pdr18*Δ cell population exhibited a higher dispersion and a higher mean value of filipin III fluorescence than the parental strain population (Fig. 5B). However, *pdr18*Δ cells grown at 40°C exhibited the highest mean values of filipin III fluorescence intensity and more than sixfold the parental strain values when grown at the same supraoptimal temperature. Total cellular ergosterol analysis, performed by two complementary methods, indicates that the *pdr18*Δ cell population exhibits a lower ergosterol content than the parental strain when grown at 30°C or 40°C and that growth at 40°C implicates a reduction in cellular ergosterol content independently of the genetic background (Fig. 5C). Although the reduction in ergosterol content caused by cultivation at 40°C is not statistically significant in the spectrophotometric‐based quantification (Fig. 5C, left panel), it was confirmed by HPLC (Fig. 5C, right panel). Despite the activation of *ERG* genes transcription in *pdr18*Δ cells, this response to counteract the deleterious effects of supra‐optimal growth temperature did not allow the full recovery of the ergosterol content, and free ergosterol precursors were significantly accumulated.

**Fig 6:**
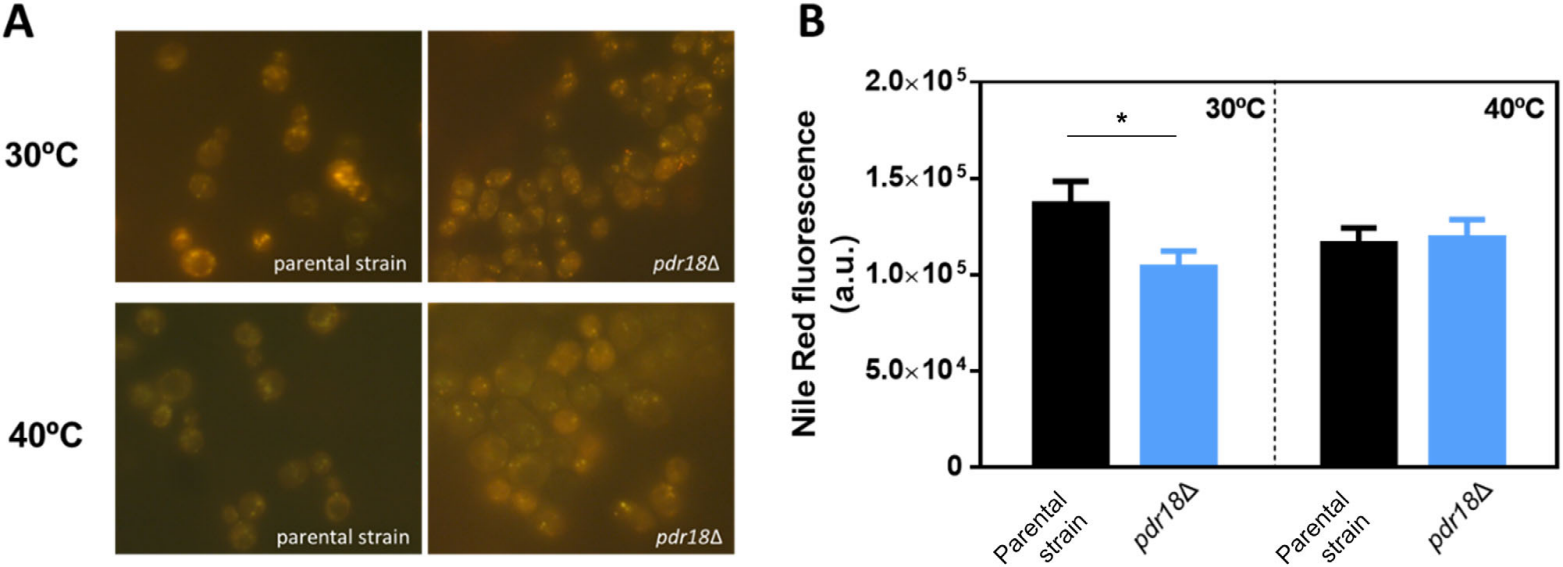
Neutral lipid quantification in parental and *pdr18*Δ strains during cultivation in YPD, at 30°C or 40°C. A. Representative images of Nile Red‐stained cells from the parental and *pdr18*Δ strains' cell populations cultivated at 30°C or 40°C; B. Comparison of Nile Red fluorescence quantification of parental and *pdr18*Δ strains' population, harvested at the indicated conditions. The data shown represent means of three independent experiments, and error bars indicate standard deviation and asterisks indicate statistical significance (*p* < 0.05; *t*‐student test).

Since the activation of the ergosterol biosynthetic pathway in *pdr18*Δ cells observed at 40°C correlated with lower ergosterol levels (Fig. 5C), the possible sequestration of sterols in lipid storage particles was examined using the fluorescent probe Nile Red that allows the quantification of the neutral storage lipids triacylglycerol and steryl esters. Since no significant differences between the parental and the *pdr18*Δ deletion mutant strains were observed in lipid classes other than sterols(Cabrito *et al*., 2011), we attribute differences registered in Nile Red fluorescence to differences in steryl esters' accumulation. Representative images of Nile Red‐stained yeast cells obtained by fluorescence microscopy are shown in Fig. 6A. However, due to the limitation of Nile Red of rapid photobleaching, the quantitative results shown were derived from fluorescence microplate reading (Fig. 6B) for all strains and conditions tested. Results suggest that *pdr18*Δ cells' population accumulated less steryl esters when compared with the parental strain when grown at 30°C. However, this difference was not observed when cells were grown at 40°C (Fig. 6B). From these results, we can conclude that when grown at 40°C, *pdr18*Δ cells exhibit lower levels of ergosterol, accumulate sterol precursors and exhibit similar levels of sterified sterols in lipid droplets.

## Discussion

Among a multitude of mechanisms that *S*. *cerevisiae* has developed to cope with the challenging conditions present in its natural environment and in industrial bioprocesses is the ability to change plasma membrane lipid and protein composition and organization (Godinho and Sá‐Correia, 2019). Plasma membrane transporters belonging to the ABC superfamily may play a dual role in yeast tolerance to chemical stresses (drugs and other xenobiotic compounds); they are hypothesized to catalyse the active efflux of the corresponding toxic compounds but some of them were implicated in lipid transport and control of plasma membrane lipidic environment (Godinho and Sá‐Correia, 2019).

In the present study, we describe, for the first time, the involvement of a putative drug‐efflux pump of the ABC superfamily in yeast thermotolerance. Pdr18 was first proposed to play a role in ergosterol transport at the yeast plasma membrane level influencing lipid membrane composition and organization (Cabrito *et al*., 2011; Godinho *et al*., 2018a). This role was found to be crucial in maintaining the physiological functioning of plasma membrane as a selective permeability barrier with a physiological electrochemical potential, especially under acetic acid‐ or 2,4‐dichlorophenoxyactetic acid (the herbicide 2,4‐D)‐induced stresses (Godinho *et al*., 2018a, Cabrito et al., 2011). Sterols are a structural element of yeast plasma membrane, conferring rigidity and counteracting its fluidification and permeabilization as the result of exposure to membrane‐disturbing agents (Douglas and Konopka, 2014; Klug and Daum, 2014). This is the case of cell incubation at supra‐optimal temperatures being its disrupting effect of plasma membrane organization especially important when conjugated with other stresses such as ethanol and acetic acid (van Uden and da Cruz Duarte, 1981; Ramos and Madeira‐Lopes, 1990) and other toxic compounds present during yeast‐mediated bioprocesses.

Genetic determinants responsible for inherent thermotolerance or, in other words, tolerance without pre‐exposure to sub‐lethal temperatures or other stresses leading to acquired thermotolerance, are of great interest in strain improvement strategies (Lindquist, 1981; Piper, 1993). Interestingly, the expression of *PDR18* is advantageous during yeast cultivation at 40°C and following rapid exposure to heat shock (30 min at 50°C) being a determinant of the inherent thermotolerance. The impact of *PDR18* expression in yeast growth at 40°C is more evident when the initial cell density is smaller. The inoculation with high cell densities is a well‐known strategy to overcome multiple physical and chemical stresses present during industrial bioprocesses (Kapu *et al*., 2013). However, important pre‐cultivation steps are required representing additional costs and manipulations. Therefore, the development of highly thermotolerant industrial strains is a desired phenotype.

For the parental strain and experimental conditions used (high initial cell density), the supraoptimal temperature tested did not have a significant effect (the maximum temperature *S*. *cerevisiae* growth for the majority of the strains is 42°C). Under these conditions, the transcription levels from genes of the ergosterol biosynthetic pathway were similar for cells grown at 30°C or at 40°C, as observed for these cells' permeability. The deletion of the *PDR18* gene, however, led to the transcriptional activation of the *ERG* genes when examined at 40°C, compared with 30°C, and with parental cells transcription levels, and *pdr18*Δ cells grown at 40°C exhibited maximum permeability values. The transcription factors Upc2 and Ecm22 are well‐known Zn(II)2‐Cys6 zinc fingers responsible for the regulation of ergosterol biosynthetic pathway genes (Vik and Rine, 2001). These transcription factors share high amino acid sequence identity and directly bind to the ergosterol molecule, sensing its intracellular level (Yang *et al*., 2015). Upc2 has been the focus of most of the regulatory studies available in the literature and the underlying regulatory mechanism is well‐described (Yang *et al*., 2015). Ergosterol binding to Upc2 maintains its cytoplasmatic localization, whereas under ergosterol depletion, the ligand‐free Upc2 moves to the nucleus leading to the transcriptional activation of ergosterol biosynthesis pathway genes (Yang *et al*., 2015). It is likely that Upc2 and Ecm22 mediate the observed transcriptional activation of the *ERG* genes examined in *pdr18*Δ cells (Davies *et al*., 2005). Considering the lower ergosterol content present in *pdr18*Δ, especially in cells grown at 40°C, the depletion of this sterol could be the trigger for *ERG* genes activation due to migration of Upc2, or both transcription factors, to the nucleus (Yang *et al*., 2015). In fact, Ecm22 was demonstrated to regulate the expression of all the *ERG* genes tested in this work and Upc2 regulates *ERG3*, *ERG9*, *ERG11* and *ERG25*, according to the information available at the YEASTRACT database (Monteiro *et al*., 2020).

Most of the ergosterol molecules in yeast cells are located at the plasma membrane and, in the case of excess of ergosterol production, an esterification reaction occurs and ergosterol is stored as steryl esters in lipid droplets (Klug and Daum, 2014). However, environmental conditions such as oxygen depletion, presence of antibiotics such as azoles and genetic manipulations can result in the blockage of the ergosterol biosynthetic pathway. The enzymes encoded by *ERG3* and *ERG5* genes catalyse two of the four last reactions of the ergosterol biosynthetic pathway and are required for the conversion of fecosterol to ergosterol. Both the individual and double deletions for these genes were found to lead to improved heat stress tolerance (Caspeta *et al*., 2014; Liu *et al*., 2017). The ergosterol precursors accumulated in these deletion mutants differ only in the number and position of the C=C double bonds (Liu *et al*., 2017). Although the accumulation of such ergosterol precursors seems advantageous in long‐term cultivation at supra‐optimal temperatures (Liu *et al*., 2017), it is likely that ergosterol precursors in the pathway earlier than fecosterol are not able to fully fulfil the ergosterol function at the plasma membrane. The results obtained in the present study led us to hypothesize that cultivation at 40°C results in the accumulation of free ergosterol precursors in *pdr18*Δ cells, while the cellular ergosterol levels remain lower and the content of esterified sterols in lipid droplets is not different from the parental strain. In a previous lipidomics analysis, the lipidome of the parental and *pdr18*Δ strains' cells grown in the absence of stress has shown that *PDR18* deletion leads to a higher relative abundance of squalene in the plasma membrane, and that this was exacerbated when the growth medium was supplemented with the weak acid herbicide 2,4‐D (Cabrito *et al*., 2011). Squalene accumulates in the cells as a result of the repression of *ERG1* transcription or the activity of the encoded enzyme that converts squalene to 2,3‐oxidosqualene. Although for the time point tested in the present study, the cellular ergosterol levels are reduced in the *pdr18*Δ mutant, the ratio of ergosterol accumulated intracellularly to the ergosterol present at the plasma membrane might be even higher than in the parental strain. A mechanism of feedback inhibition of *ERG1* expression by cytoplasmic ergosterol was previously described (Leber *et al*., 2001; Garaiová *et al*., 2014) and this regulation may be the explanation for higher squalene accumulation in the *pdr18*Δ mutant.

The presence of squalene in membranes, especially in the plasma membrane, was previously hypothesized to affect yeast growth and/or cause sensitivity to external stress (Spanova *et al*., 2012). However, the authors concluded that the ratio of ergosterol to squalene is indeed the crucial feature to define the physiological parameters of the plasma membrane (Spanova *et al*., 2012). According to our previous results, this ratio is dramatically different between *pdr18*Δ and parental strain cells (Cabrito *et al*., 2011). Interestingly, squalene is a molecule of great medical and cosmetic interest, and cells accumulating high squalene levels might be of high biotechnological potential (Gohil *et al*., 2019).

The present work represents a step forward in understanding the consequences of the deletion of Pdr18 in cellular lipid composition and, consequently, in the plasma membrane composition and biophysical properties. Under the light of our results, it can be hypothesized that the deletion of Pdr18 leads to intracellular ergosterol accumulation that is less efficiently transported at the plasma membrane level. This accumulation may trigger a feedback inhibition of *ERG1* expression, leading to the accumulation of early precursors such as squalene. These precursors are not compensatory of an ergosterol‐depleted membrane and consequently, yeast cells exhibit higher permeability and susceptibility to a multitude of environmental challenges such as supra‐optimal temperatures. Collectively, the results presented here identify Pdr18 as a thermotolerance determinant in yeast having a biological role essential to maintain plasma membrane ergosterol content and reduced non‐specific permeability during cultivation at a supraoptimal growth temperature.

## Experimental procedures

### Strains, plasmids and growth conditions

*Saccharomyces cerevisiae* parental strain BY4741 (*MATa*, *his3*Δ*1*, *leu2*Δ*0*, *met15*Δ*0*, *ura3*Δ*0*) and the derived deletion mutant *pdr18*Δ were obtained from the EUROSCARF collection (http://web.uni-frankfurt.de/fb15/mikro/euroscarf), as well as the plasmid pYCG_*PDR18*, expressing the *PDR18* gene from its natural promoter, and the corresponding cloning vector, pRS416, used for phenotypic complementation assays.

Unless stated otherwise, yeast cells were cultivated at 30°C with orbital agitation (250 rpm) in liquid minimal growth medium supplemented with the amino acids and the nucleotide to support the growth of the auxotrophic strains (MM4) or in rich YPD media. MM4 contained, per litre, 1.7 g yeast nitrogen base without amino acids and ammonium sulphate (Difco), 20 g glucose (Merck), 2.65 g (NH_4_)_2_SO_4_ (Panreac AppliChem), 20 mg l‐methionine, 20 mg l‐histidine (both from Merck), 60 mg l‐leucine and 20 mg l‐uracil (both from Sigma), adjusted to pH 4.5 with HCl (PanReac). Cells harbouring the cloning vector pRS416 or derived plasmids were obtained using the Alkali‐Cation™ Yeast Transformation Kit (MP Biomedicals) and were grown in the same medium lacking uracil supplementation (MM4‐U medium) to maintain selective pressure. YPD medium contained, per litre, 20 g glucose (Merck), 20 g bacteriological peptone and 10 g yeast extract (both from BD Biosciences), adjusted to pH 4.5 with HCl. Solid medium was prepared by the addition of 20 g L^−1^ agar (NZYTech). For all experiments in YPD, cells used as inoculum were prepared by harvesting from cultures (5000 *g*, 5 min) cultivated for 8 h in liquid YPD followed by the inoculation of fresh liquid YPD medium and growth to a standardized OD_600nm_ of 5 ± 0.5.

### Spot assays

Complementation tests were performed by spot assays with yeast cells of the parental and *pdr18*Δ strains, harbouring either the empty vector pRS416 or the recombinant plasmid pYCG_*PDR18*. Yeast cell suspensions were prepared from a mid‐exponential cell culture grown in MM4‐U medium, diluted to an OD_600nm_ of 0.25, followed by four serial dilutions of 1:5 each. These cell suspensions were plated as 4 μl spots onto the surface of MM4‐U pH 4.5 solid medium, incubated at 30°C or at 39°C. Pictures were taken after 72 h of incubation and the figures shown are representative of three independent experiments.

Yeast cell susceptibility to short‐ and long‐term exposure to supra‐optimal temperatures of growth was evaluated by spot assays in YPD medium. For short‐term exposure assays, two aliquots for both the parental and *pdr18*Δ strains were harvested by centrifugation (5000*g*, 5 min), re‐suspended in fresh YPD media and incubated at 30°C or 50°C. After an incubation time of 30 min, cell suspensions were harvested by centrifugation (5000*g*, 5 min), diluted in sterile ddH_2_O to an OD_600nm_ of 0.25, and this suspension was used to prepare four serial dilutions of 1:5 that were plated onto the surface of solid YPD media as 4 μl spots. Pictures were taken from plates incubated at 30°C for 48 h. For long‐term exposure assays, inoculum cells were harvested by centrifugation (5000*g*, 5 min), re‐suspended in sterile ddH_2_O to an OD_600nm_ of 0.25, and this suspension was used to prepare serial dilutions and spotted in YPD solid media as described before. For this assay, plates were incubated at 30°C or at 40°C, and pictures were taken after 48 h. Spot assays experiments were repeated three times as fully independent experiments.

Growth curves and cell harvesting growth curves for complementation assays were obtained by inoculating a mid‐exponential cell suspension in 50 ml flasks containing 25 ml MM4‐U pH 4.5, and incubate at 30°C or at 40°C. The starting OD_600nm_ of the cultivation media was standardized at 0.1 ± 0.5. Growth with orbital agitation was followed by measuring OD_600nm_.

Yeast cell suspensions of the parental and *pdr18*Δ strains inocula were harvested (5000*g*, 5 min) and used to inoculate fresh liquid YPD media with a standardized initial OD_600nm_ of 0.1 for microplate reader curves (FilterMax F5 Microplate Reader; Molecular Devices) and 1 for curves performed in 50‐ml Erlenmeyer flasks. Growth was performed at 30°C or at 40°C and followed by measuring OD_600nm_ for 24 h. Samples were collected from the cultivations in Erlenmeyer flasks for assessment of colony forming units per ml (CFU mL^−1^). Exponentially growing yeast cells (OD_600nm_ ≈ 5) were harvested (5000*g*, 5 min) from the curves performed in Erlenmeyer flasks at 30°C and at 40°C, for analysis of plasma membrane permeability by flow cytometry, mRNA levels of selected *ERG* genes, fluorescence microscopy imaging with filipin III and nile red, and total ergosterol quantification. Results were obtained from three independent biological replicates.

### Colony forming units

Estimation of viable cells was achieved by the determination of CFU mL^−1^ by the serial dilution method. Briefly, yeast cell suspensions were serially diluted in sterile water and 50 μl from selected dilutions were plated in solid YPD medium to obtain between 30 and 300 colonies. Plates were incubated at 30°C for 48 h and colonies were counted to assess CFUs mL^−1^. Results are means of, at least, three independent experiments.

### Flow cytometry of PI fluorescence

Analysis of PI staining by flow cytometry was conducted as described before (Davey and Hexley, 2011) with few modifications. Briefly, yeast cells corresponding to 0.2 OD_600nm_ unit in PBS were stained for 5 min with propidium iodide (PI; Sigma) to a final concentration of 6 μg ml^−1^ in Millipore Milli‐Q (0.22 mm filtered) water. The staining was performed at the same temperature of growth, not to allow the recovery of heat‐stressed cells, a phenomenon reported by Davey and Hexley (2011). Flow cytometric analyses were performed using a BD Accuri™ C6 Plus (BD Biosciences). The PI fluorescence was collected via a FL2 585/40 nm filter. All experiments were repeated using at least two biological replicates with samples being stained and analysed in duplicate. A fixed total of 50 000 events per sample were acquired using a slow flow rate (14 μl min^−1^).

### Transcription analysis of *ERG* genes

For the transcriptional analysis of *ERG* genes, exponentially growing yeast cells of both strains, grown at 30°C or 40°C, corresponding to 20 OD_600nm_ units, were immediately frozen in liquid nitrogen and total RNA was extracted by the hot phenol method (Collart and Oliviero, 1993). The reverse transcription was performed using NZY First‐Strand cDNA Synthesis Kit (NZYTech) and the primers used for the amplification of each target cDNA were designed using Primer Express (Applied Biosystems) and are shown in Supplementary Table S1. The RT‐PCR reaction was carried out using a thermal cycler block (7500 Real‐Time PCR System; Applied Biosystems) and the NZY qPCR Green Master Mix (NZYTech). The *ACT1* mRNA level was used as the internal control. The relative value obtained for the expression of each target gene in parental strain cells harvested from cultivation at 30°C was set as 1 and the remaining values were calculated relative to that. Results are means of at least two independent experiments, with three technical replicates.

### Fluorescence microscopy

Fluorescence microscopy was used to investigate free sterol content and its cellular distribution by Filipin III staining (final concentration of 0.5 μg ml^−1^) and neutral lipid content by Nile Red staining (final concentration of 2.5 μg ml^−1^). For that, yeast cells from the parental and *pdr18*Δ strains harvested from cultivation at 30°C and 40°C were re‐suspended in PBS buffer to a final OD_600nm_ of 0.5. Cells were then incubated with each probe for 20 min, in the dark, in a tube rotator (VWR) and washed twice with PBS buffer. For cell‐to‐cell analysis, fluorescence was examined with an Axioplan microscope equipped with adequate epifluorescence interface filters, obtained from Zeiss. Fluorescence emission was collected with a coupled device camera (Axiocam 503 colour; Zeiss), and the images were analysed with ZEN 2 Microscope Software (Zeiss). The exposure time was kept constant among experiments for each probe, and intensity measurements were background‐corrected. For total quantification of the population's fluorescence, cell suspensions stained with either Filipin III or Nile Red were standardized to OD_600nm_ of 0.5 and the fluorescence was measured in a Filtermax F5 Microplate Reader (Ex/Em of 360/465 nm for Filipin III and 530/625 nm for Nile Red). Results were obtained from three independent biological replicates.

### Ergosterol quantification

Total cellular ergosterol from parental and *pdr18*Δ deletion mutant strains was quantified by two complementary methods.

The spectrophotometric method was performed as previously described for *Candida* spp. isolates (Arthington‐Skaggs *et al*., 2002) and as applied to *S*. *cerevisiae* (Demuyser *et al*., 2019). The absorbance of the sterol‐containing layer was traced between 200 and 300 nm to confirm the success of the extraction by visualization of a characteristic four‐peaked curve (Arthington‐Skaggs *et al*., 2002), and OD_281.5nm_ and OD_230nm_ were used to determine ergosterol content by the equations defined before, normalized to the wet weight of the sample (Arthington‐Skaggs *et al*., 2002). Results were obtained from three independent biological replicates.

In parallel, ergosterol was extracted from a cell suspension corresponding to approximately 1000 OD_600nm_, according to the procedure described before (Santos *et al*., 2017) using cholesterol as an internal standard. The extracts were separated in a 250 mm × 4 mm C18 column (LiChroCART Purospher STAR RP‐18 end‐capped 5 μm) at 30°C. Samples were eluted in 100% methanol at a flow rate of 1 ml min^−1^. Detection of cholesterol and ergosterol was performed using an UV–vis detector set at 282 and 210 nm respectively. Under the conditions used, the retention time of cholesterol was 15 min and ergosterol was eluted at 12.5 min. Subsequent quantification of the two lipids was performed using appropriate calibration curves. The results are shown in μg of ergosterol per mg of wet cell weight. Results were obtained from three independent biological replicates.

## Supporting information


**Supplementary Table S1.** Sequences of the primers used for qRT‐PCR.
