# Supplementary material for: The ABC transporter Pdr18 is required for yeast thermotolerance due to its role in ergosterol transport and plasma membrane properties
Source: Environ Microbiol. 2020 Oct 11;23(1):69–80. doi: 10.1111/1462-2920.15253 (PMC7891575; doi:10.1111/1462-2920.15253)
Supplement: Supplementary file 1 — Supplementary Table S1. Sequences of the primers used for qRT‐PCR. [file EMI-23-69-s001.docx]

**Supplementary Table S1. Sequences of the primers used for qRT-PCR.**

| **Target gene** | **Sequence (5’-3’)** |
| --- | --- |
| **ACT1** | fw: CTCCACCACTGCTGAAAGAGAA  rev: CCAAGGCGACGTAACATAGTTTT |
| **ERG3** | fw: GCTCTGCACAAGCCTCATCA  rev: GGAAAGAATGAGATGCGAAAGG |
| **ERG9** | fw: ATCAGTCAACGTCTCCATATC  rev: GCAAACGATCTGGAGGTCAAG |
| **ERG11** | fw: CACGAATTTGTCTTCAACGCTAA  rev: AGTCAAATGAGCGTAAGCAGCTT |
| **ERG13** | fw: GATCGGTCCTGATGCTCCAA  rev: CGTAGGCGTGTTCCATGTAAGA |
| **ERG25** | fw: GCTACCCTTTCAGGTCTAGTCCAA  rev: AATGGGCGACATTTTGCAA |
